# Effects of Bcl-2/Bcl-x_L_ Inhibitors on Pulmonary Artery Smooth Muscle Cells

**DOI:** 10.3390/antiox7110150

**Published:** 2018-10-26

**Authors:** Vladyslava Rybka, Yuichiro J. Suzuki, Nataliia V. Shults

**Affiliations:** Department of Pharmacology and Physiology, Georgetown University Medical Center, Georgetown University, 3900 Reservoir Road NW, Washington, DC 20007, USA; rybkavladyslava@gmail.com (V.R.); ns1015@georgetown.edu (N.V.S.)

**Keywords:** apoptosis, Bcl-2, pulmonary hypertension, smooth muscle, vascular remodeling

## Abstract

Pulmonary arterial hypertension (PAH) is a fatal disease without satisfactory therapeutic options. By the time patients are diagnosed with this disease, the remodeling of pulmonary arteries has already developed due to the abnormal growth of pulmonary vascular cells. Therefore, agents that reduce excess pulmonary vascular cells have therapeutic potential. Bcl-2 is known to function in an antioxidant pathway to prevent apoptosis. The present study examined the effects of inhibitors of the anti-apoptotic proteins Bcl-2 and Bcl-x_L_. ABT-263 (Navitoclax), ABT-199 (Venetoclax), ABT-737, and Obatoclax, which all promoted the death of cultured human pulmonary artery smooth muscle cells. Further examinations using ABT-263 showed that Bcl-2/Bcl-x_L_ inhibition indeed promoted apoptotic programmed cell death. ABT-263-induced cell death was inhibited by antioxidants. ABT-263 also promoted autophagy; however, the inhibition of autophagy did not suppress ABT-263-induced cell death. This is in contrast to other previously studied drugs, including anthracyclines and proteasome inhibitors, which were found to mediate autophagy to induce cell death. The administration of ABT-263 to rats with PAH in vivo resulted in the reversal of pulmonary vascular remodeling. Thus, promoting apoptosis by inhibiting anti-apoptotic Bcl-2 and Bcl-x_L_ effectively kills pulmonary vascular smooth muscle cells and reverses pulmonary vascular remodeling.

## 1. Introduction

Pulmonary arterial hypertension (PAH) can affect males and females of any age, including children and young adults. Despite the availability of approved drugs, PAH remains a fatal disease without a cure [1,2]. Major pathogenic features that increase pulmonary vascular resistance include the development of vascular remodeling, in which the pulmonary artery (PA) walls are thickened and the lumens are narrowed or occluded. Increased resistance puts strain on the right ventricle, and heart failure is the major cause of death among PAH patients [3,4]. If patients are not treated, the median survival among PAH patients is 2.8 years from the time of diagnosis (three-year survival: 48%) [5,6]. Even with the currently available therapies, the prognosis remains poor; only 58–75% of patients survive for three years [7,8,9,10]. The currently available drugs primarily elicit vasorelaxation, and their ability to resolve pulmonary vascular remodeling is limited. PAH is a progressive disease, and by the time patients are diagnosed, pulmonary vascular remodeling has already occurred. Thus, therapeutic strategies that reverse pulmonary vascular remodeling should have a significant impact on health by reducing the morbidity and mortality associated with this condition.

In this regard, our laboratory has established the concept of the apoptosis-based therapy to reverse pulmonary vascular remodeling to treat PAH patients [11]. In rat models of PAH, our laboratory discovered that anti-cancer agents including the anthracycline, proteasome inhibitor, and taxane classes of drugs reversed pulmonary vascular remodeling [12,13,14]. These drugs, however, were found to also affect autophagy for the regulation of cell death. In addition to apoptosis, both anthracyclines [12] and proteasome inhibitors [13] were found to promote autophagy, and the inhibition of autophagy significantly attenuated the ability of these drugs to kill pulmonary artery smooth muscle cells (PASMCs). Thus, the mechanisms of action of these drugs involve autophagic cell death in addition to apoptotic cell death. On the other hand, a taxane anti-cancer drug, docetaxel, was found to inhibit autophagy, and the inhibition of autophagy enhanced the docetaxel-induced cell death [14]. Thus, docetaxel appears to kill pulmonary vascular cells by suppressing the survival role of autophagy since autophagy can serve as both a cell killing and cell survival mechanism [14].

Korsmeyer’s early work showed that Bcl-2 functions in an antioxidant pathway to prevent apoptosis [15]. The Bcl-2 family of anti-apoptotic proteins includes Bcl-2 and Bcl-x_L_, and various inhibitors of these proteins have been developed to promote apoptosis [16]. These drugs are expected to be purer apoptosis-inducers compared to other classes of anti-cancer drugs. Thus, in the present study, we tested the hypothesis that directly activating apoptosis using Bcl-2/Bcl-x_L_ inhibitors is sufficient to kill pulmonary artery smooth muscle cells and reverse pulmonary vascular remodeling.

## 2. Materials and Methods

### 2.1. Cell Culture Experiments

Human PASMCs purchased from ScienCell Research Laboratories (Carlsbad, CA, USA) were cultured in accordance with the manufacturer’s instructions in 5% CO_2_ at 37 °C. Passages 3–6 were used. For siRNA knockdown, cells were transfected with siRNA Transfection Reagent and test siRNA or control siRNA with a scrambled sequence (Santa Cruz Biotechnology, Dallas, TX, USA) and used for experiments 2 days later. Cells were treated with ABT-263 (Navitoclax), ABT-199 (Venetoclax), ABT-737, or Obatoclax (Selleckchem, Houston, TX, USA) dissolved in dimethyl sulfoxide (DMSO). Preliminary dose–response experiments determined the dose that produced an approximately 50% decrease in the cell number. The same dose (1 µM) was tested for all the drugs for comparison. An equal amount of DMSO (0.1%) was included as a control. The number of viable cells was determined by counting on a hemocytometer or by using a Cell Counting Kit-8 (Dojindo Molecular Technologies, Rockville, MD, USA).

### 2.2. Animal Experiments

In the present study, the SU5416/hypoxia model with pathologic features similar to those in human PAH was used [12,13,14,17,18,19,20]. Male Sprague-Dawley CD rats and male Fischer CDF rats (Charles River Laboratories, Wilmington, MA, USA) were injected with 20 mg/kg bodyweight of SU5416 subcutaneously (MedChemExpress, Monmouth Junction, NJ, USA), subjected to sustained hypoxia for 3 weeks, and then maintained in normoxia. For hypoxia, animals were placed in a chamber regulated by an OxyCycler Oxygen Profile Controller (BioSpherix, Redfield, NY, USA) to maintain 10% O_2_ with an influx of N_2_ gas [14,21]. After pulmonary hypertension and pulmonary vascular remodeling were developed, the rats were injected with ABT-263 intraperitoneally. The dose of ABT-263 used was similar to that used for other cancer drugs in our previously published studies [12,13,14].

The Georgetown University Animal Care and Use Committee approved all the animal experiments, IACUC Protocol Number is 16-015-100266. Our investigation conformed to the National Institutes of Health Guide for the Care and Use of Laboratory Animals.

### 2.3. Histological Measurements

Lung tissues were immersed in buffered 10% formalin at room temperature and were then embedded in paraffin. The paraffin-embedded tissues were cut and mounted on glass slides, and tissue sections were subjected to hematoxylin and eosin (H&E) and Verhoeff–Van Gieson stains to measure the pulmonary artery (PA) wall thickness and vessel diameter. For quantification, 13 vessels (30–100 µm diameter) were analyzed per animal. Four values of the inner and outer diameters were obtained for each vessel and the means were calculated. The wall thickness values (wall thickness divided by vessel diameter) were calculated as percentages. 

### 2.4. Western Blotting

Equal amounts of protein samples were electrophoresed through a reducing sodium dodecyl sulfate polyacrylamide gel and electroblotted onto a nitrocellulose membrane (Bio-Rad Laboratories, Hercules, CA, USA). The membrane was blocked with 5% non-fat milk or bovine serum albumin and incubated with antibodies for LC3B (Cell Signaling Technology, Danvers, MA, USA), p62 (Syd Labs, Inc., Malden, MA, USA), or glyceraldehyde-3-phosphate dehydrogenase (GAPDH; Santa Cruz Biotechnology, Dallas, TX, USA). The protein levels were detected using horseradish peroxidase-linked secondary antibodies (Bio-Rad) and the Enhanced Chemiluminescence System (GE Healthcare Life Science, Pittsburgh, PA, USA).

### 2.5. Fluorescence-Based Cell Assays for Apoptosis and Autophagy

Apoptosis was monitored using an Annexin V-enhanced green fluorescent protein (EGFP) Apoptosis Staining/Detection Kit (Abcam, Cambridge, MA, USA) that detects the translocation of the membrane phospholipid phosphatidylserine to the cell surface. 

Autophagy was assessed using an Autophagy/Cytotoxicity Dual Staining Kit (Abcam) that detects autophagic vacuoles with a fluorescent compound that is incorporated into multilamellar bodies.

### 2.6. Statistical Analysis

Means and standard errors of mean (SEM) were computed. Two groups were compared by a two-tailed Student’s *t* test, and differences between more than two groups were determined by the analysis of variance (ANOVA). A *p*-value less than 0.05 was defined as being statistically significant. 

## 3. Results

### 3.1. Bcl-2/Bcl-x_L_ Inhibitors Promote the Death of PASMCs

**Figure 1:**
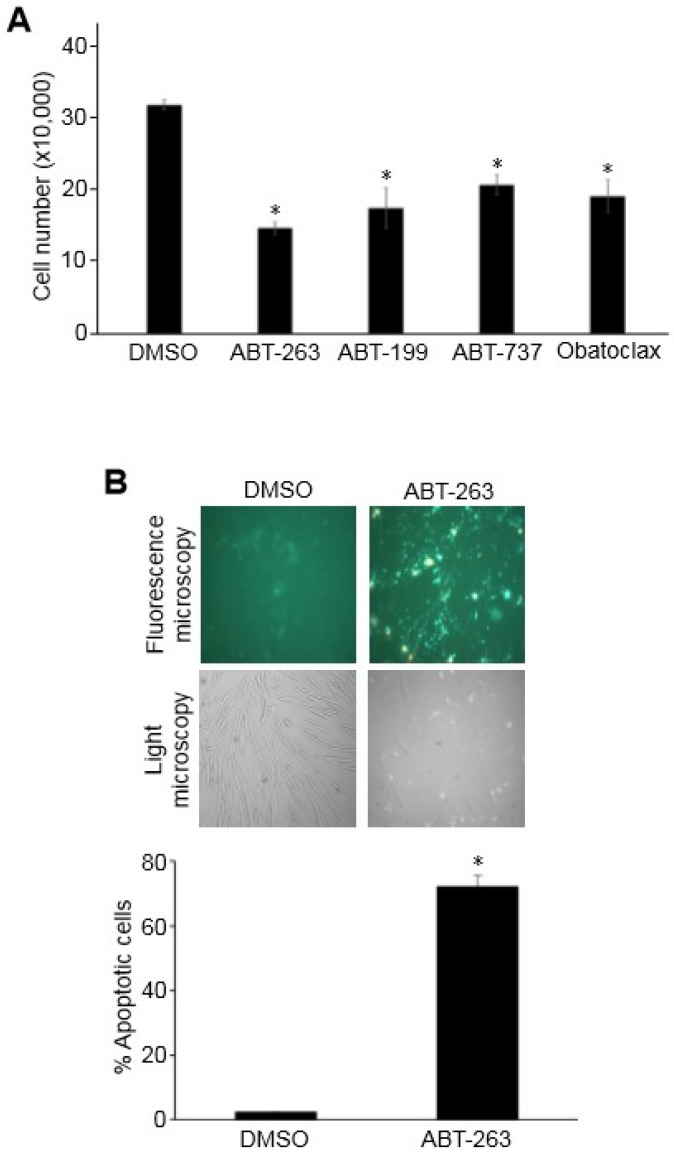
Bcl-2/Bcl-x_L_ inhibition promotes the apoptosis of human pulmonary artery smooth muscle cells (PASMCs). (**A**) Human PASMCs were treated with various Bcl-2/Bcl-x_L_ inhibitors at 1 µM for 24 h. The cell number was determined by counting on a hemocytometer. An equal amount of dimethyl sulfoxide (DMSO; 0.1%) was used as a vehicle control. The symbol * denotes that the value was significantly different from the DMSO control at *p* < 0.05 (n = 3). (**B**) Human PASMCs were treated with ABT-263 (1 µM) for 24 h. Apoptotic cells were assessed by a fluorescence-based assay that measured phospholipid phosphatidylserine by staining with an EGFP fusion of annexin V. The symbol * denotes that the value was significantly different from the DMSO control at *p* < 0.05.

**Figure 2:**
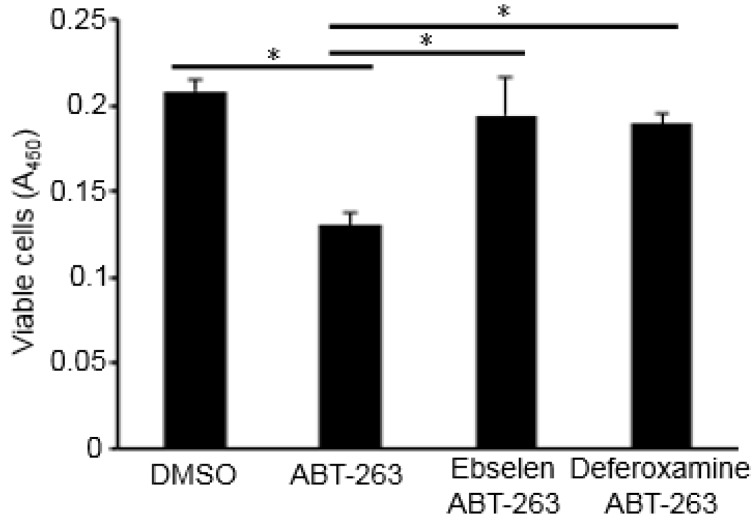
Effects of antioxidants on ABT-263-induced cell death. Human PASMCs were treated with ABT-263 (1 µM) with or without ebselen (20 µM) or deferoxamine (50 µM) for 24 h. The number of viable cells was monitored using the Cell Counting Kit-8 at absorbance 450 nm (A_450_). * denotes that the values were significantly different from each other at *p* < 0.05 (n = 8).

To investigate the effects of Bcl-2/Bcl-x_L_ inhibition on pulmonary vascular remodeling, Bcl-2/Bcl-x_L_ inhibitors including ABT-263, ABT-199, ABT-737, and Obatoclax were studied in cultured human PASMCs. All tested drugs that were dissolved in DMSO at 1 µM significantly decreased the number of cells when compared to control cells treated with an equal amount of DMSO (Figure 1A). As expected, the inhibition of anti-apoptotic proteins Bcl-2/Bcl-x_L_ promoted apoptotic programmed cell death as indicated by a fluorescence-based assay that measures phospholipid phosphatidylserine by staining with an EGFP fusion of annexin V (Figure 1B). The Cell Counting Kit-8 assay, which determines the number of living cells by measuring the amount of formazan dye generated by dehydrogenase also showed that the treatment of PASMCs with 1 µM ABT-263 for 24 h resulted in a significant decrease in viable cells (Figure 2). 

Since Bcl-2 has been shown to promote antioxidant pathways during its anti-apoptotic activity [15], the inhibition of Bcl-2 and Bcl-x_L_ may decrease the antioxidant activity, thus resulting in cell death. Indeed, replacement with antioxidants such as ebselen (a selenium-containing glutathione peroxidase mimetic that scavenges hydrogen peroxide) or deferoxamine (an iron chelator that inhibits the Fenton reaction) effectively inhibited ABT-263-induced cell death (Figure 2).

### 3.2. Autophagy Does Not Mediate Bcl-2/Bcl-x_L_-Inhibitor-Induced Cell Death

**Figure 3:**
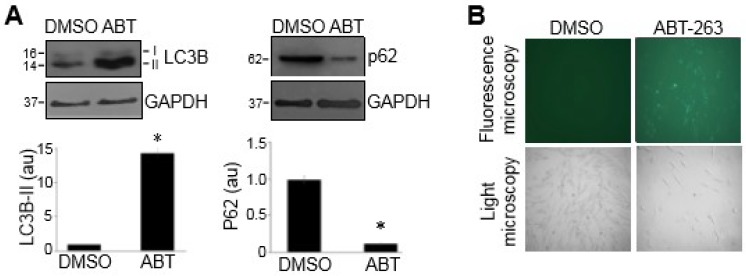
ABT-263 promotes autophagy. Human PASMCs were treated with DMSO (0.1%) or ABT-263 (1 µM) for 24 h. (**A**) Cell lysates were subjected to Western blotting to monitor the LC3B-II and p62 levels. The bar graph represents the mean ± standard error of the mean (SEM) of the protein levels determined by densitometry expressed in an arbitrary unit (au). * denotes that the value was significantly different from the DMSO control at *p* < 0.05 (n = 3). (**B**) Autophagy was assessed by the detection of autophagic vacuoles with a green fluorescent compound that was incorporated into multilamellar bodies.

**Figure 4:**
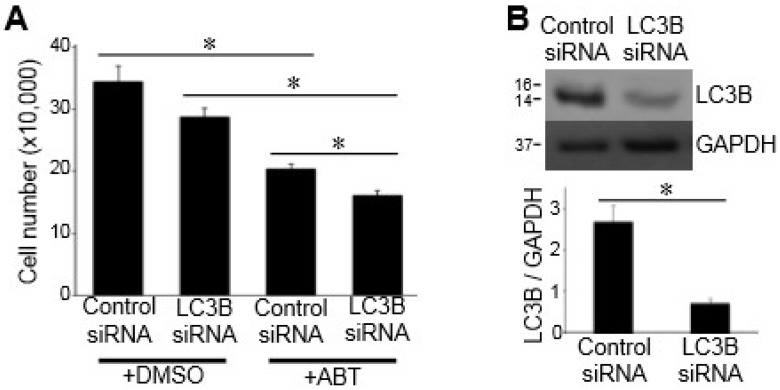
The role of autophagy in the ABT-263-induced death of PASMCs. (**A**) Cells were transfected with siRNA for microtubule-associated proteins 1A/1B light chain 3B (LC3B) or control siRNA. Cells were then treated with DMSO or ABT-263 (1 µM), and the cell number was counted. (**B**) Western blotting results demonstrating the extent of siRNA knockdown of LC3B expressed as the ratio of LC3B to glycealdehyde 3-phosphate dehydrogenase (GAPDH) proteins. * denotes that values were significantly different from each other at *p* < 0.05 (n = 4).

Our laboratory previously found that anti-cancer drugs such as daunorubicin, bortezomib, carfilzomib, and MG-132 killed PASMCs, in part through autophagy-mediated cell death [12,13]. These drugs were found to promote autophagy and inhibit autophagy-blocked cell death. Similarly, ABT-263 was also found to promote autophagy, as indicated by the increased LC3B-II and decreased p62 (as monitored by Western blotting as shown in Figure 3A) and by detecting autophagic vacuoles with a fluorescent compound that is incorporated into multilamellar bodies (Figure 3B). However, in contrast with our earlier observations regarding other cell-killing drugs [12,13], ABT-263-induced cell death was not attenuated by inhibiting autophagy through knocking down an essential autophagy protein LC3B (Figure 4), suggesting that ABT-263-induced cell death does not mediate autophagic cell death.

### 3.3. Bcl-2/Bcl-x_L_-Inhibition Reverses Pulmonary Vascular Remodeling in Rats

To test whether Bcl-2/Bcl-x_L_-inhibition can reverse pulmonary vascular remodeling in vivo, the effects of ABT-263 on rats with already-developed pulmonary vascular remodeling were studied. To induce PAH and pulmonary vascular remodeling, rats were injected with SU5416 (a vascular endothelial growth factor receptor inhibitor) and subjected to sustained hypoxia using a well-established protocol that elicits pathological features similar to those in human PAH [12,13,14,17,18,19,20]. While Sprague-Dawley rats have been the most-commonly used strain for this model [12,13,14,17,18,19], a recent study demonstrated that Fischer CDF rats can exhibit a more severe condition [20]; the present study confirmed the findings using both of these strains.

**Figure 5:**
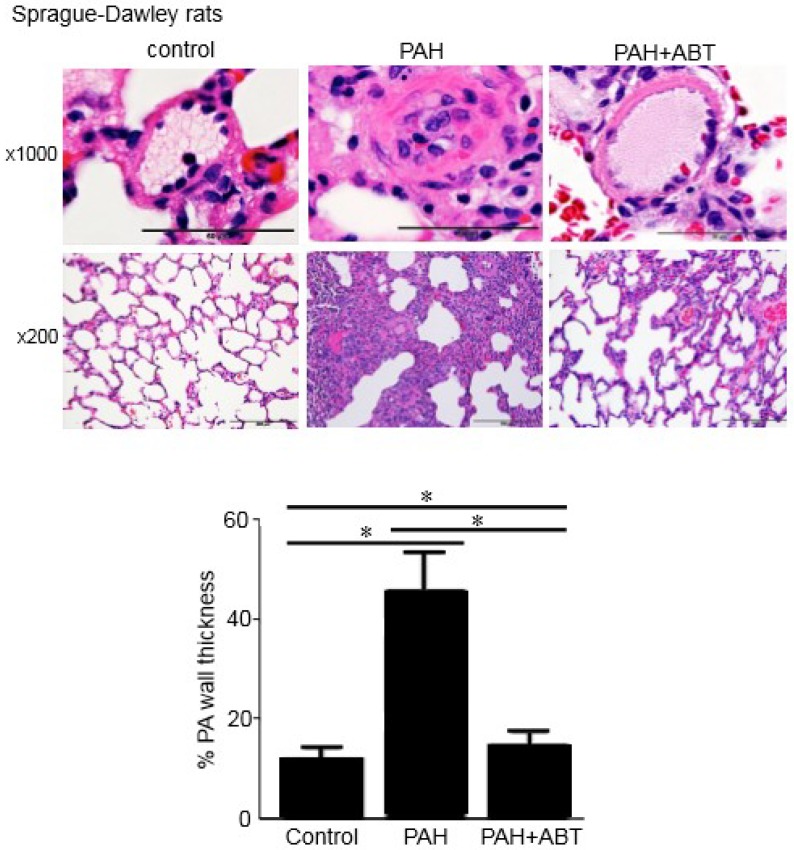
ABT-263 reverses pulmonary artery (PA) remodeling in Sprague-Dawley rats treated with SU5416/hypoxia. Sprague-Dawley rats were treated with SU5416, subjected to sustained hypoxia for three weeks, and then maintained in normoxia for five weeks. After pulmonary vascular remodeling was developed, ABT-263 (5 mg/kg body weight) was then injected four times over a two-week period. Lung tissues were immersed in buffered 10% formalin and embedded in paraffin for haematoxylin and eosin (H&E) staining. Representative images at ×1000 and ×200 magnifications are shown. The bar graph represents means ± SEM of % PA wall thickness (n = 5). * denotes that the values were significantly different from each other at *p* < 0.05.

**Figure 6:**
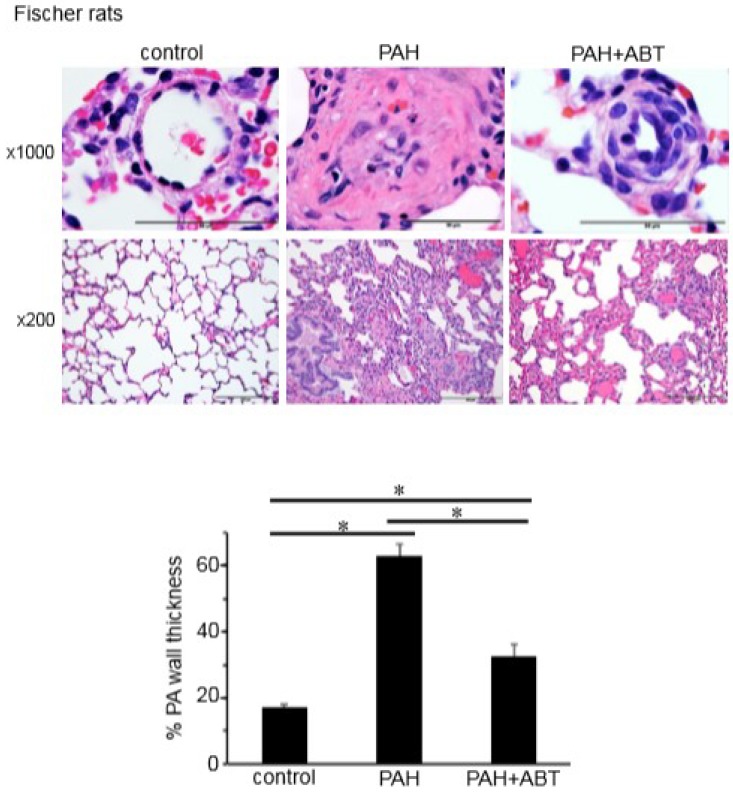
ABT-263 reverses PA remodeling in Fischer rats treated with SU5416/hypoxia. Fischer (CDF) rats were treated with SU5416 and sustained hypoxia (three weeks) and then maintained in normoxia. After pulmonary vascular remodeling was developed, ABT-263 was then injected. The lungs were harvested, immersed in buffered 10% formalin and embedded in paraffin for H&E staining. Representative images at ×1000 and ×200 magnifications are shown. The bar graph represents means ± SEM of % PA wall thickness (n = 5). * denotes that the values were significantly different from each other at *p* < 0.05.

The H&E stain results shown in Figure 5 and Figure 6 (magnification ×1000) demonstrate the remodeling of the small pulmonary vessels in the two strains of rats with PAH compared to the normal small PAs in the control rats. The thicknesses of all three layers of the blood vessel—the intima, the media, and the adventitia—were significantly increased. Histology data from the Sprague-Dawley and Fischer rats with PAH showed complete occlusions of small PAs by concentric endothelial cell proliferation, hypertrophy and hyperplasia of smooth muscle in the media layer, and an increase in collagen deposition around the vessels. The ABT-263 treatment effectively reversed the pulmonary vascular remodeling. The vessel wall thickness was reduced in both strains of rats, and the lumens of the PAs were significantly greater (Figure 5 and Figure 6, magnification ×1000).

Figure 5 and Figure 6 (magnification ×200) show the lung sections of the Sprague-Dawley and Fischer rats in the control, PAH and after ABT-263 treatment. In addition to pulmonary vascular remodeling in PAH, the histology results revealed atelectasis and the emphysematous expansion of the alveoli as well as a thickened alveolar septa due to the inflammatory infiltration. A perivascular inflammatory infiltrate around the vessels was also observed. The treatment with ABT-263 normalized the remodeled lung structure in PAH: the inflammatory infiltrate in the alveolar septa was significantly decreased, and most of the lumens of alveoli were filled with air.

## 4. Discussion

The present study was designed to provide further information regarding the cell death mechanisms in pulmonary vascular cells in order to facilitate the development of effective apoptosis-based therapies to reverse pulmonary vascular remodeling for the treatment of PAH patients [11]. We have previously reported that anti-tumor drugs capable of promoting the apoptosis of cultured pulmonary vascular smooth muscle cells, including the anthracycline and proteasome inhibitor class of drugs, reversed pulmonary vascular remodeling in the in vivo models of PAH [12,13]. The ability of these drugs to reduce pulmonary vascular thickening was specific to the pulmonary vessels of animals with pulmonary vascular remodeling, while normal control pulmonary vessels were unaffected, demonstrating the selectivity of this approach to killing cells of the diseased pulmonary vasculature. Mechanistically, in the pulmonary vascular cells it was interesting to note that both classes of these drugs not only promoted apoptosis but also mediated autophagic cell death [12,13]. 

Bcl-2 and Bcl-x_L_ played major roles in regulating programmed cell death by serving as anti-apoptotic proteins. These molecules can be inhibited by the Bcl-2 homology 3 (BH3)-only proteins of the Bcl-2 family, resulting in apoptosis [22]. In this regard, BH3 mimetics have been developed to inhibit the activity of Bcl-2 and Bcl-x_L_ in order to promote apoptosis to kill unwanted cancer cells [23]. Since PAH has similar features as cancer, possessing abnormally grown, untoward cells that reduce and/or occlude pulmonary vessels, the use of these agents may have a therapeutic benefit for the treatment of PAH.

Here, we reported that in cultured human PASMCs, the BH3 mimetic drugs that inhibit anti-apoptotic Bcl-2 and Bcl-x_L_—including ABT-263 (Navitoclax), ABT-199 (Venetoclax), ABT-737 and Obatoclax—promoted programmed cell death. BH3-mimetic-induced death appears to be oxidant-dependent as it was blocked by antioxidants, confirming the antioxidant role of the Bcl-2 family of anti-apoptotic proteins [15]. While our previous studies showed that cell death drugs including anthracyclines and proteasome inhibitors promoted both apoptotic and autophagic cell death [12,13], inhibiting autophagy did not attenuate the capacity of a BH3 mimetic to promote cell death. Thus, Bcl-2/Bcl-x_L_ inhibitors more strictly utilize apoptosis and do not mediate other types of programmed cell death, especially autophagic cell death. 

In the in vivo models of PAH, ABT-263 administration after the vascular remodeling caused the reversal of the remodeled lesions with significantly increased lumen sizes. Thus, the inhibition of Bcl-2 and Bcl-x_L_ by BH3 mimetics can effectively reverse pulmonary vascular remodeling. Sprague-Dawley rats are the most commonly utilized strain for the SU5416/hypoxia model of PAH that exhibits a vascular pathology similar to humans [12,13,14,17,18,19]. Fischer CDF rats have more recently been shown to promote more severe PAH [20]. The present study showed the beneficial effects of ABT-263 in both of these strains of rats. In these experiments, ABT-263 was administered after the severe pulmonary vascular remodeling was promoted. Thus, the improvement of the pathology must involve the elimination of vascular structural cells such as smooth muscle cells. Our cell culture results are consistent with the idea that ABT-263 directly targets smooth muscle cells. However, these experiments cannot rule out the possibility that other cell types were targeted by ABT-263 to reverse pulmonary vascular remodeling.

## 5. Conclusions

In summary, targeting the antioxidant pathway of the Bcl-2 family of apoptotic proteins is an effective way to kill unwanted pulmonary vascular cells without mediating the autophagy process. Further work is needed to address questions regarding which BH3 mimetics are effective in vivo, what may be the most beneficial timing, dosing and the route of administration, as well as whether these drugs exert side effects, in order to potentially use this class of drugs for the treatment of PAH. Understanding the death and survival mechanisms of pulmonary vascular cells of remodeled vessels should also provide information that will be useful for designing new strategies to reverse pulmonary vascular remodeling.
